# The Influence of Parental Control on Emotional Eating Among College Students: The Mediating Role of Emotional Experience and Regulation

**DOI:** 10.3390/nu17172756

**Published:** 2025-08-26

**Authors:** Leran Wang, Yuanluo Jing, Shiqing Song

**Affiliations:** 1 School of Psychology, Shaanxi Normal University, Xi’an 710062, China; suzizhan69@gmail.com; 2 Faculty of Education and Society, University College London, London WCE1 6BT, UK; jingyluo@126.com

**Keywords:** parental control, emotional eating, negative emotions, emotion regulation strategies

## Abstract

**Background**: Excessive parental control has been found to be associated with an increasing risk of emotional eating in children, yet the potential moderating role of emotion regulation abilities remains unclear. This study investigated the relationships between different types of parental control and emotional eating, as well as the mediating effects of specific emotion regulation strategies and negative emotions. **Methods**: A cross-sectional online survey was conducted with 1167 Chinese college students (62.5% females, age: 20.23 ± 1.50 years) recruited via social media. Participants completed the Parental Control Scale, Emotion Regulation Questionnaire, Depression Anxiety Stress Scales, and Dutch Eating Behavior Questionnaire. Data were analyzed using SPSS and PROCESS (Model 81), with BMI, age, and gender controlled as a covariate. Mediation effects were tested using the 95% bias-corrected bootstrap confidence intervals (based on 5000 samples). **Results**: The results indicate that (1) both parental behavioral control and psychological control were significantly positively correlated with emotional eating, with effect sizes ranging from small to moderate; (2) anxiety and stress in negative emotions partially mediate the relationship between the two dimensions of parental control and emotional eating, while depression did not serve as a mediator in this relationship; (3) expression suppression and stress chain-mediated between the two dimensions of parental control and emotional eating; expression suppression and anxiety chain-mediated between parental psychological control and emotional eating. **Conclusions**: Higher parental control is associated with increased emotional eating behaviors in children. Anxiety, stressful emotions, and expressive suppression play significant roles. These findings suggest new interventions to reduce emotional eating and associated overweight risks in college students.

## 1. Introduction

### 1.1. Emotional Eating

Increasing academic demands, competitive employment prospects, and lifestyle changes in contemporary China have been associated with elevated negative emotions among college students [1,2,3]. At the same time, negative emotions trigger a higher risk of emotional eating and weight gain [4,5,6]. Emotional eating is consuming food in response to negative emotions (e.g., anxiety, stress, or depression), causing unnecessary intake when not hungry [7,8]. Individuals typically prefer high-calorie, high-fat, and high-sugar foods to temporarily relieve negative emotions [9,10]. In addition to contributing to being overweight, emotional eating may also undermine adolescents’ self-esteem and emotion regulation abilities [11].

Emotional eating occurs more frequently among overweight and obese individuals than in normal-weight groups [12,13], contributing to weight-related health issues. Although research focused on overweight and obese populations [4,14], normal-weight individuals are also affected. Emotional eating is considered a learned behavior [15,16], implying that children and adolescents with established tendencies may persist into college years. Recent European studies highlight the impact of lifestyle on stress-related eating. Specifically, Moscatelli et al. found that a nutrition-education intervention for Italian undergraduates demonstrated how campus health behaviors influence emotional eating [17]. These findings suggest that emotional eating serves not only as a coping mechanism but also as a modifiable component of broader lifestyle patterns within university environments, which present potential risks to students’ mental and physical health. Consequently, this study investigates determinants of emotional eating among college students.

### 1.2. Parental Control and Emotional Eating

While most of the previous research on emotional eating focused on individual factors such as negative emotional experiences or gender differences, the development of emotional eating behaviors is not only influenced by personal reasons but also equally by family factors. According to the ecosystem theory proposed by Bronfenbrenner, the family belongs to the most central “microsystem” and is the closest environment that interacts directly with the individual, and its impact on the individual’s development is direct, far-reaching, and continuous [18]. Parenting styles significantly influence children’s emotions and behaviors [19,20,21], with parental control emerging as a pivotal dimension [22,23].

Parental control refers to parental strategies for managing and regulating children, comprising two dimensions [24]: behavioral control (establishing rules, restrictions, and monitoring activities) [25] and psychological control (intruding into the child’s psychological sphere to undermine autonomy via interference, guilt induction, and love withdrawal) [26]. Previous research has focused on the impact of parental control on children’s emotions and found that excessive behavioral and psychological control by parents can lead to negative emotions such as social anxiety [27,28] and non-suicidal self-injury behaviors [29]. Studies in Western cultural contexts suggest that higher parental control increases the likelihood of emotional eating in children [30]. In Chinese cultural settings, the model of the parent–child relationship differs from that of the West, with parental control generally being stronger in East Asia [31]. Evidence regarding the relationship between parental control and emotional eating from the Chinese context remains limited.

In addition, although behavioral control and psychological control are closely related, they differ in their impact on children [32,33]. Previous findings have shown that behavioral control positively affects children’s behavioral norms, increasing resilience, and reducing delinquent behavior [34,35,36], while psychological control leads to decreased psychological safety, increased levels of depression, lower quality of friendships, and poor adaptation [37,38,39,40]. Furthermore, evidence suggests that the effect of behavioral control may differ across developmental stages. Research indicates that heightened behavioral control exerted on individuals in older age groups may increase their children’s susceptibility to emotional eating [30]. However, the participants in this study were college students, and it remains uncertain whether early adulthood populations would exhibit similar patterns to those observed in previous studies.

In summary, researchers generally agree that behavioral control contributes to the development of good behavioral patterns in children and is a positive parenting style, while psychological control may hurt children. It is, therefore, necessary to further explore the differences in the effects of different types of control on children’s emotional eating behavior.

### 1.3. The Relationship Between Negative Emotions, Parental Control, and Emotional Eating

High levels of parental control are closely associated with the emergence of negative emotions in children. Previous research has found that high levels of psychological control exerted by parents can lead to negative emotions such as anxiety, depression, and stress in their children [27,28], and may even trigger non-suicidal self-injurious behaviors in children [29]. Studies related to eating have also indicated a significant positive correlation between negative emotions associated with emotional eating and parental control [28].

Negative emotions such as stress and anxiety are key triggers for emotional eating and are commonly considered as core variables in related studies. Research indicates that negative emotions heighten food sensitivity [5], subsequently increasing food intake—particularly among overweight/obese individuals relative to normal-weight counterparts [41]. Consistent results have also been found among various groups, including adult women, adolescents, and college students. Results consistently indicate that individuals with higher levels of depression, anxiety, and stress exhibit a greater tendency toward emotional eating [4,42,43]. These findings suggest that negative emotions are significant triggers for emotional eating, with women being more susceptible to stress-induced emotional eating compared to men [44,45].

In summary, this study posits that excessively high levels of parental control lead to negative emotions in children, which, in turn, is a key influencing factor for emotional eating. Therefore, it is hypothesized that negative emotions serve as a mediating factor between parental control and emotional eating.

### 1.4. The Relationship Between Emotion Regulation Strategies, Parental Control, and Emotional Eating

Individuals use emotion regulation strategies to identify and regulate specific emotions, thereby enhancing desired emotional experiences and expressions. According to Gross, the two types of widely accepted emotion regulation strategies currently include expressive suppression and cognitive reappraisal [46,47]. Expressive suppression inhibits emotional expression (e.g., suppressing anger/sadness) and correlates with elevated depression and anxiety [48]. Conversely, cognitive reappraisal reinterprets emotional events (e.g., reframing failure as learning) and is associated with greater life satisfaction and reduced depression/anxiety [48,49,50].

A large number of studies demonstrate that different emotion regulation strategies can influence the emergence of negative emotions. Individuals who tend to use cognitive reappraisal strategies experience and express more positive emotions and fewer negative emotions, while individuals who use expressive suppression strategies may experience and express more negative emotions and fewer positive emotions [48,51]. This indicates a close relationship between the choice of emotion regulation strategies and the emergence of negative emotions.

Previous studies have found that parental behavioral control reduces emotional regulation difficulties in children, playing a positive role [52,53]. In contrast, parental psychological control is negatively associated with children’s adaptive emotion regulation, and where heightened levels correlate with greater regulation difficulties [54]. Additionally, excessive parental psychological control promotes children’s emotional suppression while reducing their utilization of strategies such as cognitive reappraisal [55,56], indicating that the type of parental control also affects children’s emotion regulation strategies.

Research has shown that emotion regulation is negatively correlated with the tendency to eat emotionally [57], with difficulties in emotion regulation increasing the risk of eating problems [58]. In addition, empirical research has found that poor emotion regulation strategies negatively mediate the relationship between maternal parenting behaviors and children’s emotional eating when children are rejected and ostracized by their mothers [59]. Moreover, empirical research has found that when children experience rejection and exclusion by their mothers, maladaptive emotion regulation strategies negatively mediate the relationship between maternal parenting behavior and children’s emotional eating [60]. This evidence suggests that emotion regulation strategies can influence the relationship between parent–child relationships and emotional eating.

In summary, emotion regulation strategies are a key factor influencing emotional eating. Within the parenting relationship, the type and level of parental control also impact children’s emotional regulation abilities and strategies. Therefore, the present study suggests that emotional regulation strategy may be an important mediator that influences the relationship between parental control and emotional eating.

Based on the above research, this paper proposes the following three questions:

1. Both parental behavioral control and psychological control are significantly associated with emotional eating, but do their effects align?
2. Do negative emotions mediate the relationship between parental control and emotional eating?
3. Do emotion regulation strategies mediate the relationship between parental control and emotional eating?

This study provides empirical evidence for the field of emotional eating and provides a theoretical basis for family factors that influence emotional eating.

## 2. Materials and Methods

### 2.1. Measures

Data from participants was distributed and collected via an online questionnaire platform (Wenjuanxing, https://www.wjx.cn/, accessed on 11 December to 18 December 2023). Participants were contacted via social media from various regions; they were college students. This questionnaire included the Parental Control Scale, the Emotion Regulation Questionnaire, the Depression Anxiety Stress Scales, and the Dutch Eating Behavior Questionnaire. All personal information was anonymized to ensure confidentiality. Moreover, each participant signed an informed consent form before testing and had the option to withdraw from the study at any time. At the end of the survey, participants received a remuneration of CNY 10 (approximately equal to USD 1.4).

### 2.2. Participants

This study recruited 1522 college participants. A preliminary screening revealed that 355 participants were excluded for failing to pass the attention check, consistency validation, patterned responding, or response time. The screening method is as follows: (1) Attention checks using instructional items (e.g., “select ‘never’”), and excluded participants with ≥3 non-compliant responses. Based on this method, we excluded 118 people; (2) consistency validation via semantically identical pairs, and eliminated cases with >3 contradictory answers. Based on this method, we excluded 96 people; (3) patterned responding followed the criteria proposed by Berinsky et al., we excluded participants with ≥5 consecutive identical ratings [61]. Based on this method, we excluded 77 people; (4) response times <100 s were excluded as implausible, accounting for the cognitive demands of the composite instrument. Based on this method, we excluded 64 people. Ultimately, 1167 valid data sets were retained, including 438 males (37.5%) and 729 females (62.5%). The average age of the participants was 20.23 years (SD = 1.50), and the average body mass index (BMI) was 20.77 (SD = 2.80). Gender, age, and BMI were statistically controlled as covariates in the analyses. This study was ethically approved by the Human Experiment Ethics Committee of the College of Psychology, Shaanxi Normal University (HR2025-04-01). All participants signed the informed consent form approved by the committee. All subjects received some payment upon task completion.

### 2.3. Materials

#### 2.3.1. Parental Control

The study used the Parental Control Scale developed by Wang et al. to measure the extent of parental control experienced by the participants [36]. This scale comprises two subscales: behavioral control and psychological control, with 34 items. The behavioral control subscale consists of 16 items, primarily measuring two aspects: parental inquiries (e.g., “My parents ask me about my extracurricular activities”) and limitation of freedom (e.g., “My parents require that I obtain their permission before going out again after returning home from school”). The psychological control subscale contains 18 items divided into three dimensions: inducing guilt (e.g., “When I don’t do things the way my parents want, they tell me they are disappointed in me”), withdrawal of love (e.g., “When I do something against my parents’ wishes, they tell me I am not a good family member”), and exerting authority (e.g., “My parents told me that what they wanted me to do was what was best for me and that I shouldn’t have to question these things.”). The scale employs a 5-point scoring system, ranging from “strongly disagree” to “strongly agree,” with scores from 1 to 5. The average score of all items is calculated, with higher scores indicating stronger parental control over the child. Good internal consistency reliability has been verified in a school-based Chinese sample [62], with Cronbach’s α coefficients of 0.903 and 0.926 for the behavioral control subscale and psychological control subscale, respectively. The Cronbach’s α coefficient for this measurement was 0.95. The structural validity was good, with χ^2^/*df* = 3.523, RMSEA = 0.047, CFI = 0.956, TLI = 0.944, RFI = 0.924, and NFI = 0.940. All constructs showed satisfactory composite reliability (CR > 0.70); the psychological control’s CR was 0.952; the behavioral control’s CR was 0.852. For average variance extracted (AVE), the psychological control construct reached 0.531, and the behavioral control construct yielded an AVE of 0.303.

#### 2.3.2. Emotion Regulation Strategies

The study utilized the Emotion Regulation Questionnaire (ERQ) developed by Gross, which includes two dimensions, cognitive reappraisal and expressive suppression, comprising 10 items [51]. The cognitive reappraisal dimension consists of 6 items (e.g., “When I want to feel certain positive emotions (such as happiness or joy), I change my perspective on the situation”), while the expressive suppression dimension consists of 4 items (e.g., “I do not express my emotions”). The scale employs a 7-point Likert scoring system, ranging from “strongly disagree” to “strongly agree,” with scores from 1 to 7. Higher scores indicate a greater frequency of the corresponding strategy’s use. Prior validation with Chinese adolescents [63] confirmed strong internal consistency for both cognitive reappraisal (α = 0.84) and expressive suppression (α = 0.81). The Cronbach’s α coefficient for this scale was 0.74. The structural validity was good, with χ^2^/*df* = 3.344, RMSEA = 0.045, CFI = 0.977, TLI = 0.967, RFI = 0.954, and NFI = 0.967. All constructs showed satisfactory composite reliability (CR > 0.70); the cognitive reappraisal’s CR was 0.799; the expressive suppression’s CR was 0.791. For average variance extracted (AVE), the cognitive reappraisal construct reached 0.401, and the behavioral control construct yielded an AVE of 0.487.

#### 2.3.3. Negative Emotion

The study employed the Depression Anxiety Stress Scales (DASS) developed by Antony et al. [64], which consists of 21 items across three dimensions: depression (7 items, e.g., “I seem not to be able to experience any pleasurable or enjoyable feelings”); stress (7 items, e.g., “I find it hard to relax”); and anxiety (7 items, e.g., “I often feel dry in the mouth”). The scale uses a 4-point Likert scoring system, ranging from “strongly disagree” to “strongly agree,” with scores from 1 to 4. Higher scores indicate more severe negative emotions. The scale has been proven to have good reliability in a Chinese sample [65], with Cronbach’s α coefficients of 0.94 for the overall scale. The Cronbach’s α coefficient for this scale was 0.95. The structural validity was good, with χ^2^/*df* = 3.484, RMSEA = 0.046, CFI = 0.972, TLI = 0.961, RFI = 0.947, and NFI = 0.962. All constructs showed satisfactory composite reliability (CR > 0.70); the stress’s CR was 0.874; the anxiety’s CR was 0.833; the depression’s CR was 0.854. For average variance extracted (AVE), the stress construct reached 0.499; the anxiety construct yielded an AVE of 0.418; the behavioral control construct was 0.460.

#### 2.3.4. Emotional Eating

The emotional eating dimension of the Dutch Eating Behavior Questionnaire (DEBQ), developed by Van Strien et al., was used to measure the level of emotional eating in subjects [66]. This dimension comprises 13 reformulated items (e.g., “Do you feel the urge to eat when you are angry?”). A 5-point Likert scoring system was used, ranging from “strongly disagree” to “strongly agree,” with scores from 1 to 5. Higher scores indicate a greater tendency towards emotional eating. The subscales showed good reliability in Chinese youth [67], with Cronbach’s α coefficients of 0.93. The structural validity was good, with χ^2^/*df* = 3.444, RMSEA = 0.046, CFI = 0.990, TLI = 0.982, RFI = 0.975, and NFI = 0.987. The discriminant validity average variance extracted (AVE) = 0.560, and the composite reliability (CR) = 0.942.

### 2.4. Data Analysis

The data were statistically analyzed using SPSS 26.0 and PROCESS v4.0. The descriptive and correlation analyses were conducted in SPSS 26.0, while the PROCESS (version 4.0, Model 81) was used to run a serial mediation analysis to determine whether the negative emotions and regulation strategies mediated the relationship between parental control and emotional eating. To test the direct, indirect, and total effects of parental control on emotional eating, we employed bootstrap sampling (5000 iterations) with bias-corrected 95% confidence intervals, reducing Type I error risks.

Self-report data carried common method variance (CMV) risks attributable to emotional or social desirability effects. Therefore, we employed the unmeasured latent method construct test to assess CMV. The results show that the differences between the no-method-factor model (M1) and the method-factor model (M2) were ΔCFI < 0.1, ΔTLI < 0.1, and ΔRMSEA < 0.05. As can be seen, CMV did not have much impact on the results of this study.

Multicollinearity tests were also conducted in this study. The correlation values (all r < 0.8), variance inflation factors (VIF) (all VIF < 4), and tolerances (all tolerances > 0.2) indicate that there are no multicollinearity issues in this study.

## 3. Results

### 3.1. Descriptive Statistics and Correlation Analysis

Table 1 presents variable means, standard deviations, and Pearson correlation coefficients. The correlation analysis suggests that emotional eating is significantly correlated with expressive suppression, depression, anxiety, stress, psychological control, and behavioral control, but not correlated with cognitive reappraisal. The correlation coefficient is shown in Table 1. Cognitive reappraisal is significantly correlated with depression, anxiety, stress, and behavioral control, but not correlated with emotional eating, expressive suppression, or psychological control.

### 3.2. Mediation Effect Test

This study used Model 81 from the PROCESS v4.0 macro in SPSS 26.0 software to test the mediation effect between emotion regulation strategies and negative emotions. Because of the different effects of the two parental controls and the two modes of emotion regulation, they were modeled separately, and a total of four models were developed for this experiment, all of which controlled for BMI, gender, and age as covariates. According to the method of Bootstrap provided by Hayes, it was also validated to analyze the mediating role of emotion regulation styles and negative emotions between parental control and emotional eating. Indirect effects were tested using bootstrap resampling (5000 samples) with bias-corrected 95% confidence intervals (default in PROCESS v4.0) [68]. All β coefficients are standardized.

#### 3.2.1. The Relationship Between Parental Behavioral Control and Emotional Eating Mediated by Expressive Suppression and Negative Emotions (Model 1)

**Figure 1:**
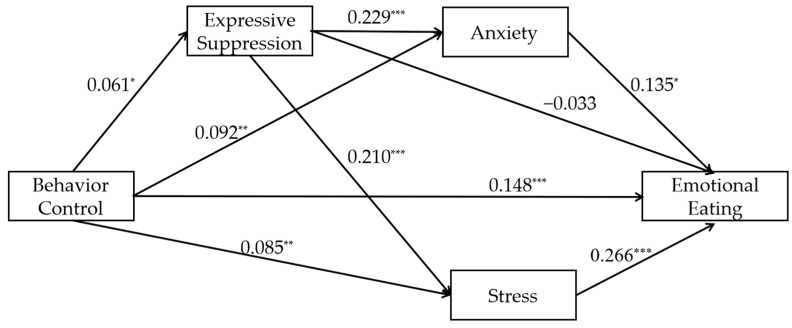
Path diagram of the standardized mediating model of expressive suppression and negative emotions between behavioral control and emotional eating (Model 1). Note: * *p* < 0.05, ** *p* < 0.01, *** *p* < 0.001. All β coefficients are standardized.

The path coefficients for Model 1 are shown in Figure 1, and the effect sizes and confidence intervals for each mediator are detailed in Table 2. If the confidence interval does not cross 0, it suggests that the mediating effect is significant. The results show that behavioral control was significantly positively associated with emotional eating (β = 0.148, t = 5.524, *p* < 0.001). The results of the mediation effect tests were as follows: (1) In the mediation effect test of expressive suppression, behavioral control was significantly positively associated with expressive suppression (β = 0.061, t = 2.080, *p* = 0.038), but expressive suppression was not significantly associated (β = −0.033, t = −1.238, *p* = 0.216; Ind1). Ind1 mediating effect is not significant, indicating that expressive suppression may not mediate the relationship between behavioral control and emotional eating; (2) in the mediation effect test of negative emotions, behavioral control was positively associated with anxiety significantly (β = 0.092, t = 3.246, *p* = 0.001) and significantly predicted stress positively (β = 0.085, t = 2.984, *p* = 0.003). Anxiety significantly predicted emotional eating positively (β = 0.128, t = 2.320, *p* = 0.021; Ind2) and stress significantly predicted emotional eating positively (β = 0.259, t = 4.875, *p* < 0.001; Ind3). The significant mediating effects of Ind2 and Ind3 align with the possibility that anxiety and stress mediate the relationship between behavioral control and emotional eating; (3) in the chained mediation effect of expressive suppression and negative emotions, expressive suppression significantly predicted anxiety positively (β = 0.229, t = 8.084, *p* < 0.001; Ind4), and significantly predicted stress positively (β = 0.210, t = 7.343, *p* < 0.001; Ind5). The nonsignificant mediating effect of Ind4 suggests that the expressive suppression and anxiety may not mediate the relationship between behavioral control and emotional eating. In contrast, the significant effect of Ind5 indicates that expressive suppression and stress can mediate the relationship between behavioral control and emotional eating. The effect strength of all pathways is small to moderate. The final R^2^ value for emotional eating was 0.178, the R^2^ value for expressive suppression was 0.004, the R^2^ value for anxiety was 0.067, and the R^2^ value for stress was 0.055.

According to the model results, the direct effect of the independent variable on the dependent variable is 0.148 (95% CI [0.095, 0.200], 79.57% of total effect). The total indirect effect is 0.038 (95% CI [0.016, 0.062], 20.430% of total effect), indicating that the mediating variable plays a certain role in the mechanism. When examining the paths separately (see Table 2), Ind2 (0.012, accounting for 6.452%) and Ind3 (0.023, accounting for 12.366%) were both significant but had small effects, while Ind1 (−0.002, accounting for −1.075%) and Ind4 (0.002, accounting for 1.075%) were not significant. Ind5 (0.003, accounting for 1.613%) is close to significant but has a very small effect. Overall, direct pathways predominantly drive model effects, while indirect contributions remain limited in magnitude. Since none of the mediating models for depression were significant, their specific details were not discussed in this section.

#### 3.2.2. The Relationship Between Parental Behavioral Control and Emotional Eating Mediated by Cognitive Reappraisal and Negative Emotions (Model 2)

**Figure 2:**
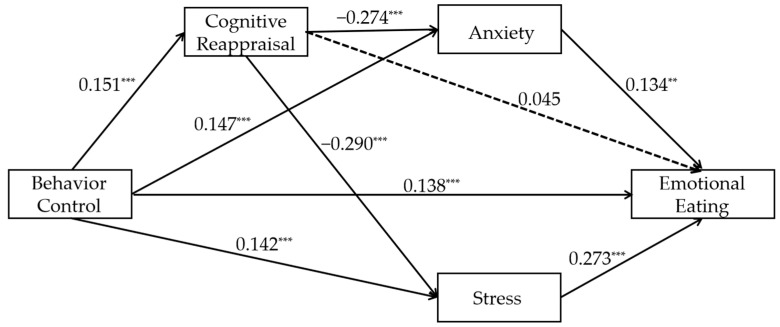
Path diagram of the standardized mediating model of cognitive reappraisal and negative emotions between behavioral control and emotional eating (Model 2). Note: ** *p* < 0.01, *** *p* < 0.001. The dashed line indicates that the two variables are not correlated with each other. All β coefficients are standardized.

The path coefficients for Model 2 are shown in Figure 2, and the effect sizes and confidence intervals for each mediator are detailed in Table 3. The results show that behavioral control was significantly positively associated with emotional eating (β = 0.138, t = 5.083, *p* < 0.001). The results of the mediation effect tests were as follows: (1) In the mediation effect test for cognitive reappraisal, behavioral control was positively associated with cognitive reappraisal significantly (β = 0.143, t = 5.216, *p* < 0.001), whereas the positive predictive effect of cognitive reappraisal on emotional eating was not significant (β = 0.047, t = 1.652, *p* = 0.099; Ind1), the mediating effect of Ind1 is not significant, indicating that cognitive reappraisal may not mediate the relationship between behavioral control and emotional eating; (2) in the mediation effect test for negative emotions, behavioral control was positively associated with anxiety significantly (β = 0.147, t = 5.196, *p* < 0.001). and stress (β = 0.141, t = 5.008, *p* < 0.001). Anxiety was positively associated with emotional eating significantly (β = 0.123, t = 2.233, *p* = 0.026; Ind2), and stress was positively associated with emotional eating significantly (β = 0.264, t = 4.959, *p* < 0.001; Ind3). The significant mediating effects of Ind2 and Ind3 indicating that anxiety and stress mediate the relationship between behavioral control and emotional eating; (3) in the chain mediation effect of cognitive reappraisal and negative emotions, cognitive reappraisal was negatively associated with anxiety significantly (β = −0.274, t = −9.618, *p* < 0.001; Ind4), and was negatively associated with stress significantly (β = −0.290, t = −10.200, *p* < 0.001; Ind5). Ind4 and Ind5 show significant mediation, indicating that cognitive reappraisal of the anxiety/stress chain effects may connect behavioral control to emotional eating. The effect strength of all pathways is small to moderate. The R^2^ value for emotional eating was 0.179, the R^2^ value for cognitive reappraisal was 0.031, the R^2^ value for anxiety was 0.087, and the R^2^ value for stress was 0.092.

According to the model results, the direct effect of the independent variable on the dependent variable is 0.138 (95% CI [0.085, 0.192], 74.194% of the total effect). The total indirect effect is 0.048 (95% CI [0.022, 0.074], 25.806% of total effect), indicating that the mediating variable contributes to the mechanism. When examining the paths separately (see Table 3), Ind2 (0.020, accounting for 10.753%) and Ind3 (0.039, accounting for 20.968%) were significant, while Ind1 (0.007, accounting for 3.763%), Ind4 (−0.006, accounting for −3.226%), and Ind5 (−0.012, accounting for −6.452%) were not significant. Similar to Model 1, direct pathways predominantly drive model effects, while indirect contributions remain limited in magnitude. Depression still has no significant impact.

#### 3.2.3. The Relationship Between Parental Psychological Control and Emotional Eating Mediated by Cognitive Reappraisal and Negative Emotions (Model 3)

**Figure 3:**
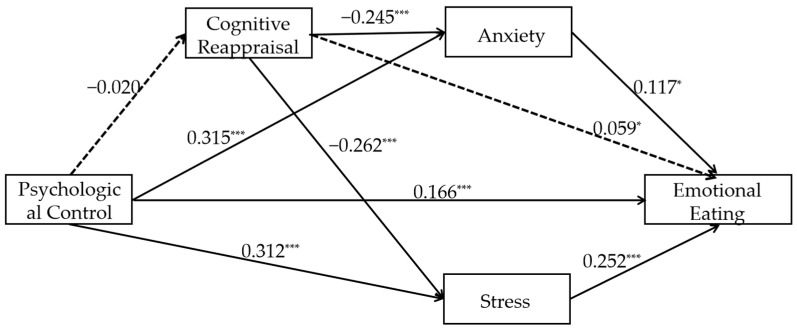
Path diagram of the standardized mediating model of cognitive reappraisal and negative emotions between psychological control and emotional eating (Model 3). Note: * *p* < 0.05, *** *p* < 0.001. The dashed line indicates that the two variables are not correlated with each other. All β coefficients are standardized.

The path coefficients for Model 3 are shown in Figure 3, and the effect sizes and confidence intervals for each mediator are shown in Table 4. The results show that psychological control was significantly positively associated with emotional eating (β = 0.167, t = 5.873, *p* < 0.001). The results of the mediation effect tests were as follows: (1) In the mediation effect test for cognitive reappraisal, the negative association between psychological control and cognitive reappraisal was not significant (β = −0.018, t = −0.673, *p* = 0.501), while cognitive reappraisal was positively associated with emotional eating significantly (β = 0.058, t = 2.069, *p* = 0.039; Ind1), the mediating effect of Ind1 is not significant, indicating that cognitive reappraisal may not mediate the relationship between psychological control and emotional eating; (2) in tests of mediating effects of negative emotions, psychological control was positively associated with anxiety significantly (β = 0.315, t = 11.731, *p* < 0.001), and stress (β = 0.312, t = 11.676, *p* < 0.001). Anxiety was positively associated with emotional eating significantly (β = 0.121, t = 2.208, *p* = 0.027; Ind2), and stress was positively associated with emotional eating significantly (β = 0.256, t = 4.816, *p* < 0.001; Ind3). Significant mediation by Ind2 and Ind3 suggests that anxiety and stress may mediate the association of psychological control with emotional eating; (3) among the chain-mediated effects of cognitive reappraisal and negative affect, cognitive reappraisal was significantly negatively associated with anxiety (β = −0.250, t = −9.109, *p* < 0.001; Ind4), and stress (β = −0.262, t = −9.758, *p* < 0.001; Ind5). Ind4 and Ind5 show no significant mediation, suggesting that cognitive reappraisal with anxiety/stress does not substantially mediate the pathway from psychological control to emotional eating. The effect strength of all pathways is small to moderate. The final R^2^ value for emotional eating was 0.185, the R^2^ value for expressive suppression was 0.008, the R^2^ value for anxiety was 0.165, and the R^2^ value for stress was 0.170.

According to the model results, the direct effect of the independent variable on the dependent variable is 0.162 (95% CI [0.108, 0.216], 58.696% of the total effect). The total indirect effect is 0.113 (95% CI [0.089, 0.146], 40.942% of the total effect), indicating that the mediating variable makes a relatively significant contribution to the mechanism of action. Specifically, the paths Ind2 (0.036, accounting for 13.043%) and Ind3 (0.077, accounting for 27.899%) were significant; Ind1 (−0.001, accounting for −0.362%), Ind4 (0.001, accounting for 0.362%), and Ind5 (0.001, accounting for 0.362%) were all insignificant. Overall, direct effects remain primary, but indirect effects—particularly through Ind2 and Ind3—are equally significant and require consideration. Depression still has no significant impact.

#### 3.2.4. The Relationship Between Parental Psychological Control and Emotional Eating Mediated by Expressive Suppression and Negative Emotions (Model 4)

**Figure 4:**
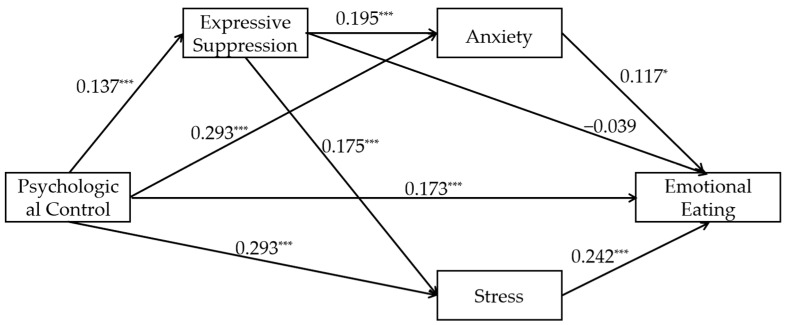
Path diagram of the standardized mediating model of expressive suppression and negative emotions between psychological control and emotional eating (Model 4). Note: * *p* < 0.05, *** *p* < 0.001. All β coefficients are standardized.

The path coefficients for Model 4 are shown in Figure 4, and the effect sizes and confidence intervals for each mediator are shown in Table 5. The results show that psychological control was significantly positively associated with emotional eating (β = 0.174, t = 6.156, *p* < 0.001). The results of the mediation effect tests were as follows: (1) In the mediation effect test for expressive suppression, psychological control was positively associated with expressive suppression significantly (β = 0.137, t = 4.731, *p* < 0.001), the association between expressive suppression and emotional eating was not significant (β = −0.036, t = −1.350, *p* = 0.177; Ind1), while the mediating effect of Ind1 is not significant, indicating that expressive suppression may not mediate the relationship between psychological control and emotional eating; (2) in the mediation effect test for negative emotions, psychological control was positively associated with anxiety significantly (β = 0.293, t = 10.669, *p* < 0.001), and stress (β = 0.293, t = 10.628, *p* < 0.001). Anxiety was positively associated with emotional eating significantly (β = 0.127, t = 2.317, *p* = 0.021; Ind2), and stress was positively associated with emotional eating significantly (β = 0.250, t = 4.708, *p* < 0.001; Ind3). Significant mediation by Ind2 and Ind3 suggests that anxiety and stress may mediate the association of psychological control with emotional eating; (3) in the chain mediation effect of expressive suppression and negative emotions, expressive suppression was significantly associated with anxiety (β = 0.195, t = 7.107, *p* < 0.001; Ind4), and stress (β = 0.175, t = 6.332, *p* < 0.001; Ind5). The significant indirect effects of Ind4 and Ind5 suggest that chains of expressive suppression related to anxiety and stress may mediate the relationship between psychological control and emotional eating. The effect strength of all pathways is small to moderate. The final R^2^ value for emotional eating was 0.183, the R^2^ value for expressive suppression was 0.019, the R^2^ value for anxiety was 0.143, and the R^2^ value for stress was 0.132.

According to the model results, the direct effect of the independent variable on the dependent variable is 0.169 (95% CI [0.115, 0.223], 61.232% of the total effect), indicating a moderate effect. The total indirect effect is 0.106 (95% CI [0.079, 0.136], 38.406% of the total effect), indicating a moderate effect, suggesting that the mediating variable makes a significant contribution to the mechanism of action. Specifically, the paths Ind2 (0.034, accounting for 12.319%), Ind3 (0.069, accounting for 25.000%), Ind4 (0.003, accounting for 1.087%), and Ind5 (0.006, accounting for 2.174%) all exhibit significant positive effects, with Ind2 and Ind3 showing more pronounced effects. Ind1 (−0.005, accounting for −1.812%) is not significant, indicating the presence of a statistical suppression pattern, though its influence is minor. Overall, direct effects remain dominant, and multi-mediator pathways (particularly Ind2/Ind3 mediation) constitute essential components. Depression still has no significant impact.

## 4. Discussion

### 4.1. Parental Control and Emotional Eating

The results of this study indicated that stronger parental control was associated with a higher tendency for children to engage in emotional eating. This finding partially confirmed previous conclusions. However, we also discovered differing results. While earlier researchers suggested that behavioral control can regulate children’s behavior and provide positive guidance [34,35,36], our results reveal a positive correlation between the level of behavioral control and children’s emotional eating. This suggested that the greater the parental behavioral control, the higher the levels of emotional eating tendencies exhibited by the children. Based on this result, we speculate that it may be closely related to the age stage of the participants. For children and adolescents who are in the period of behavioral norm shaping, behavioral control can have more positive influences. However, for college students, who are in an early stage of adulthood and have developed a greater sense of self-esteem and independence, higher levels of parental behavioral control may be associated with greater resistance, which could contribute to more negative emotions. Previous research has also supported this speculation. Snoek et al. found that both the high psychological control experienced by individuals in the lower age range and the high behavioral control experienced by individuals in the upper age range may be positively associated with the occurrence of emotional eating [30]. Additionally, this may be related to cultural background; past studies have predominantly focused on Western cultures. In contrast, in traditional Chinese parenting styles, parents tend to have high levels of control over their children’s psychological and behavioral aspects, which may be linked to stronger negative emotions regarding both psychological and behavioral control. However, from the results, we were able to find higher correlations between psychological control and all three negative emotions, as well as emotional eating tendencies, compared to behavioral control. This indicated that psychological control in parenting appears to produce more negative effects on children’s emotions as well as emotional eating behaviors.

### 4.2. Mediating Effects of Negative Emotions and Emotion Regulation Strategies

This study found that anxiety and stress partially mediated the relationship between parental control and emotional eating in college students, which partially validated previous research. Specifically, higher levels of parental behavioral and psychological control were linked to increased anxiety and stress, and these emotions may increased risk of emotional eating behaviors. This finding aligned with earlier studies that indicated parental control increases children’s anxiety and stress [27,28], as well as research showing that individuals with higher levels of anxiety and stress tend to exhibit a greater tendency for emotional eating [4,12,42,43]. This study revealed that parental control was an important factor in enhancing children’s negative emotions and was also a significant factor in inducing emotional eating. This may be due to excessive behavioral and psychological control from parents, which can lead children to feel that their parents have excessively high expectations for them, or it may cause college students, who are entering early adulthood, to feel oppressed, resulting in anxiety and stress. To vent these emotions, they may choose emotional eating as a means of relief, thereby promoting emotional eating behaviors.

This study further found that the two types of emotion regulation strategies did not directly mediate the relationship between parental control and emotional eating. Instead, they partially mediated this relationship in a chain mediation manner through anxiety and stress.

For the model of parental behavioral control and emotional eating, expression suppression and stress acted as chain mediators, which was consistent with previous findings that individuals who employed expression suppression emotion regulation strategies were likely to experience and express more negative emotions and fewer positive emotions [48,51]. An interesting finding of this study was the positive correlation between behavioral control and expressive suppression. We believe this may be because, under excessive behavioral control from parents, children experience more negative emotions, while their emotional expression is suppressed. As a result, the accumulating pressure has no outlet for release and may be related to children’s higher emotional eating as a way to relieve excessive stress. Additionally, cognitive reappraisal also mediated the relationship with anxiety and stress in a chain manner; however, these two paths exhibited a suppressing effect. We believe this could be due to the presence of other influential variables that have not been identified among these variables, resulting in a suppressing impact. According to previous research, behavioral control is considered a positive parenting approach [34,35,36], and, thus, behavioral control may be associated with the use of cognitive reappraisal as a positive emotion regulation strategy. Cognitive reappraisal has been recognized as an effective emotion regulation strategy [48,49,50] and effectively reduces anxiety and stress. However, the alleviating effect of cognitive reappraisal might not completely offset the negative impact of parental behavioral control. Therefore, even though cognitive reappraisal reduces negative emotions, it may still be insufficient to mitigate children’s emotional eating behaviors. There may also be deeper influential factors affecting this relationship, and, thus, we will further explore this in future research.

In the model of parental psychological control and emotional eating, expressive suppression had a partial mediating effect on anxiety and stress, which was also consistent with previous research. The higher the level of parental psychological control, the more likely children are to tend to suppress their emotions during emotion regulation [55,56]. Additionally, expressive suppression increases children’s feelings of anxiety and stress [48,51], and the accumulation of these anxiety and stress emotions can prompt children to adopt emotional eating as a strategy to alleviate their negative emotions [4,42,43]. This confirms that adverse parenting practices encourage children to use ineffective emotion regulation strategies, leading to the accumulation of negative emotions, which, in turn, increase emotional eating behaviors.

Although previous studies have confirmed a strong predictive relationship between parental control and children’s emotional eating, the underlying psychological mechanisms remain unclear. Therefore, this study focuses on exploring the impact mechanisms of different types of parental control on children’s emotional eating, while also expanding the research on family factors related to emotional eating in college students. The findings provide empirical evidence for the field of emotional eating research.

At the practice level, this study also provided some insights for parents, educators, and emotional eating intervention research. Parents appropriately reduce their behavioral and psychological control over their college-aged children for the benefit of their children’s physical and mental health, which also helps to reduce their children’s emotional eating behaviors. Secondly, the chain-mediating effects of expression suppression and negative emotions also suggested to parents and educators that guiding their children to use proper emotion regulation may help to reduce their children’s negative emotions and further reduce their children’s emotional eating as a maladaptive eating behavior.

### 4.3. Research Limitations and Future Directions

This study has shortcomings that need to be improved upon in future studies. First, observed suppression effects in the parental behavioral control–emotional eating mediation model (with cognitive reappraisal and negative emotions as mediators) indicate unmeasured confounding variables (e.g., self-identity, body image). We can incorporate these variables into the model in further study. Additional covariate factors, including socioeconomic status and parental education, may further influence this framework. Therefore, we also need to control these extraneous variables. Secondly, there are limitations in participant recruitment and data collection processes. The participants were all recruited in China. The samples with a single cultural background affected the cross-cultural applicability of the results. Moreover, the online recruitment approach leads to increased data exclusion rates and potential challenges in maintaining sample representativeness. Third, demographic imbalances were observed in gender and age distribution: there is a higher proportion of female participants compared to male participants, and the sample consisted mainly of young undergraduates. Although gender and age were controlled as covariates, these imbalances may still affect result representativeness. Fourth, the cross-sectional design employed in this study limits causal inference among parental control, emotion regulation, negative emotions, and emotional eating behavior, as concurrent measurement precludes establishing temporal precedence. Longitudinal designs are needed to verify causal pathways and directional sequences in the future.

## 5. Conclusions

This study investigated the associations between parental control and emotional eating among Chinese college students, examining the mediating roles of negative emotions and emotion regulation strategies. Within the context of Chinese culture, both parental behavioral control and psychological control were positively associated with students’ emotional eating behaviors. Higher parental control was also related to heightened anxiety and stress levels, which subsequently increased the risk of emotional eating. Regarding emotion regulation strategies, expressive suppression functioned as a sequential mediator alongside anxiety and stress. Stronger parental behavioral control corresponded to greater use of expressive suppression, which was associated with heightened stress and, afterwards, higher risk of emotional eating. Similarly, higher psychological control is related to increased expressive suppression, connecting to accumulated negative emotions and emotional eating. These findings suggest potential intervention targets, such as screening students who report high perceived parental control for anxiety and stress, and providing training to enhance emotional regulation abilities.

## Figures and Tables

**Table 1:** The descriptive statistical results of each variable and the correlations between the variables (N = 1167).

Variable	M	SD	1	2	3	4	5	6	7
1. Emotional eating	2.468	0.865	—						
2. Cognitive reappraisal	5.213	0.767	−0.038	—					
3. Expressive suppression	3.870	1.204	0.063 *	−0.004	—				
4. Depression	1.772	0.579	0.329 **	−0.321 **	0.259 **	—			
5. Anxiety	1.929	0.572	0.365 **	−0.254 **	0.235 **	0.812 **	—		
6. Stress	2.098	0.611	0.387 **	−0.270 **	0.217 **	0.798 **	0.844 **	—	
7. Psychological control	2.761	0.876	0.284 **	−0.024	0.138 **	0.326 **	0.321 **	0.317 **	—
8. Behavior control	3.100	0.694	0.185 **	0.148 **	0.059 *	0.070 *	0.106 **	0.098 **	0.485 **

**Note:** * *p* < 0.05, ** *p* < 0.01.

**Table 2:** Indirect effect sizes and confidence intervals for each path in Model 1.

Path	Effect Value	Effect Size (%)	SE	95%CI	
				Lower Limit	Upper Limit
Direct effect	0.148	79.570	0.027	0.095	0.200
Ind1	−0.002	−1.075	0.002	−0.007	0.002
Ind2	0.012	6.452	0.007	0.002	0.027
Ind3	0.023	12.366	0.009	0.007	0.043
Ind4	0.002	1.075	0.001	−0.000	0.005
Ind5	0.003	1.613	0.002	0.000	0.008
Total indirect effect	0.038	20.430	0.012	0.016	0.062
Total effect	0.186		0.029	0.129	0.242

**Note: Ind1:** Behavioral Control → Expressive Suppression → Emotional Eating (not significant); **Ind2:** Behavioral Control → Anxiety → Emotional Eating (significant); **Ind3:** Behavioral Control → Stress → Emotional Eating (significant); **Ind4:** Behavioral Control → Expressive Suppression → Anxiety → Emotional Eating (not significant); **Ind5:** Behavioral Control → Expressive Suppression → Stress → Emotional Eating (significant).

**Table 3:** Indirect effect sizes and confidence intervals for each path in Model 2.

Path	Effect Value	Effect Size (%)	SE	95%CI	
				Lower Limit	Upper Limit
Direct effect	0.138	74.194	0.027	0.085	0.192
Ind1	0.007	3.763	0.005	−0.002	0.017
Ind2	0.020	10.753	0.009	0.004	0.039
Ind3	0.039	20.968	0.011	0.019	0.063
Ind4	−0.006	−3.226	0.003	−0.011	−0.001
Ind5	−0.012	−6.452	0.004	−0.020	−0.006
Total indirect effect	0.048	25.806	0.013	0.022	0.074
Total effect	0.186		0.029	0.129	0.242

**Note: Ind1:** Behavioral Control → Cognitive Reappraisal → Emotional Eating (not significant); **Ind2:** Behavioral Control → Anxiety → Emotional Eating (significant); **Ind3:** Behavioral Control → Stress → Emotional Eating (significant); **Ind4:** Behavioral Control → Cognitive Reappraisal → Anxiety → Emotional Eating (significant); **Ind5:** Behavioral Control → Cognitive Reappraisal → Stress → Emotional Eating (significant).

**Table 4:** Indirect effect sizes and confidence intervals for each path in Model 3.

Path	Effect Value	Effect Size (%)	SE	95%CI	
				Lower Limit	Upper Limit
Direct effect	0.162	58.696	0.028	0.108	0.216
Ind1	−0.001	−0.362	0.002	−0.006	0.002
Ind2	0.036	13.043	0.017	0.003	0.070
Ind3	0.077	27.899	0.018	0.043	0.114
Ind4	0.001	0.362	0.001	−0.001	0.003
Ind5	0.001	0.362	0.002	−0.003	0.006
Total indirect effect	0.113	40.942	0.015	0.089	0.146
Total effect	0.276		0.028	0.222	0.330

**Note: Ind1:** Psychological Control → Cognitive Reappraisal → Emotional Eating (not significant); **Ind2:** Psychological Control → Anxiety → Emotional Eating (significant); **Ind3:** Psychological Control → Stress → Emotional Eating (significant); **Ind4:** Psychological Control → Cognitive Reappraisal → Anxiety → Emotional Eating (not significant); **Ind5:** Psychological Control → Cognitive Reappraisal → Stress → Emotional Eating (not significant).

**Table 5:** Indirect effect sizes and confidence intervals for each path in Model 4.

Path	Effect Value	Effect Size (%)	SE	95%CI	
				Lower Limit	Upper Limit
Direct effect	0.169	61.232	0.028	0.115	0.223
Ind1	−0.005	−1.812	0.004	−0.014	0.003
Ind2	0.034	12.319	0.016	0.002	0.067
Ind3	0.069	25.000	0.018	0.002	0.067
Ind4	0.003	1.087	0.002	0.000	0.007
Ind5	0.006	2.174	0.002	0.002	0.011
Total indirect effect	0.106	38.406	0.015	0.079	0.136
Total effect	0.276		0.028	0.222	0.330

**Note: Ind1:** Psychological Control → Expressive Suppression → Emotional Eating (not significant); **Ind2:** Psychological Control → Anxiety → Emotional Eating (significant); **Ind3:** Psychological Control → Stress → Emotional Eating (significant); **Ind4:** Psychological Control → Expressive Suppression → Anxiety → Emotional Eating (significant); **Ind5:** Psychological Control → Expressive Suppression → Stress → Emotional Eating (significant).

## Data Availability

The original contributions presented in the study are included in the article; further inquiries can be directed to the corresponding author.
